# Identification and validation of *N* ^7^‐methylguanosine‐associated gene NCBP1 as prognostic and immune‐associated biomarkers in breast cancer patients

**DOI:** 10.1111/jcmm.18067

**Published:** 2023-12-10

**Authors:** Jianrong Li, Lin Zheng, Liying Song, Zhuanxia Dong, Wenqi Bai, Liqiang Qi

**Affiliations:** ^1^ Department of General Surgery Sciences Shanxi Province Cancer Hospital/Shanxi Hospital Affiliated to Cancer Hospital, Chinese Academy of Medical Sciences/ Cancer Hospital Affiliated to Shanxi Medical University Taiyuan China; ^2^ Department of Vascular Surgery The Second Hospital of Shanxi Medical University Taiyuan China; ^3^ Thyroid Surgery Department First Hospital of Shanxi Medical University Taiyuan China; ^4^ Gastroenterology Shanxi Province Cancer Hospital/Shanxi Hospital Affiliated to Cancer Hospital, Chinese Academy of Medical Sciences/Cancer Hospital Affiliated to Shanxi Medical University Taiyuan China; ^5^ Department of Breast Surgery, National Cancer Center/National Cancer Clinical Medical Research Center/Cancer Hospital Chinese Academy of Medical Sciences Beijing China

**Keywords:** breast cancer, differentially expressed genes, immune, *N* ^7^‐methylguanosine, NCBP1

## Abstract

We intend to evaluate the importance of *N* ^7^‐methylguanosine (m7G) for the prognosis of breast cancer (BC). We gained 29 m7G‐related genes from the published literature and among them, 16 m7G‐related genes were found to have differential expression. Five differentially expressed genes (CYFIP1, EIF4E, EIF4E3, NCBP1 and WDR4) were linked to overall survival. This suggests that m7G‐related genes might be prognostic or therapeutic targets for BC patients. We put the five genes to LASSO regression analysis to create a four‐gene signature, including EIF4E, EIF4E3, WDR4 and NCBP1, that divides samples into two risky groups. Survival was drastically worsened in a high‐risk group (*p* < 0.001). The signature's predictive capacity was demonstrated using ROC (10‐year AUC 0.689; 10‐year AUC 0.615; 3‐year AUC 0.602). We found that immune status was significantly different between the two risk groups. In particular, NCBP1 also has a poor prognosis, with higher diagnostic value in ROC. NCBP1 also has different immune states according to its high or low expression. Meanwhile, knockdown of NCBP1 suppresses BC malignancy in vitro. Therefore, m7G RNA regulators are crucial participants in BC and four‐gene mRNA levels are important predictors of prognosis. NCBP1 plays a critical target of m7G mechanism in BC.

## 1 INTRODUCTION

One of the most prevalent cancers in females, breast cancer (BC) has a devastating impact on patients' quality of life.^1^ Early clinical indications of the illness are not evident, and tiny lesions are difficult to detect in clinical breast examinations, breast ultrasounds and mammograms. Also, recent studies have been searching for predictive and prognostic markers but they are not yet available for clinical use in BC patients.^2^ Thus, most women do not receive prompt treatment until more visible clinical signs appear, missing the window of opportunity for surgery altogether.^3^ Especially for triple‐negative BC, the incidence of brain metastasis has been estimated between 25% and 46%.^4^ Prediction of prognostics is difficult because of sophisticated detection procedures with low specificity. Furthermore, given the restricted therapy options for BC,^5^ there is a pressing need for new prognostic models to be developed.

*N* ^7^‐methylguanosine (m7G) exists in the internal sites of transfer RNAs and rRNA which are the most significantly edited RNA species in cells, including m7G. More and more studies have proved m7G's correlation with different diseases in humans, in particular carcinoma.^6^, ^7^, ^8^ Human malignancies are linked to epigenetic alterations in RNA that aren't properly controlled. m7G has been identified to take a significant role in BC in previous research, and several genes, such as NSUN2^9^ and the METTL1 gene,^10^ are known to adversely regulate m7G. METTL1 methyltransferase mediates m7G methylation within let‐7e miRNA, activating the let‐7e miRNA/HMGA2 axis which was linked with a poor prognosis. Meanwhile, other m7G‐related genes, such as DCP2^10^ and DCPS, may cause illness by committing the transcript to destruction. However, it is unclear if these m7G‐regulated genes are associated with the prognosis of BC samples.

To carry out this research, we used TCGA databases to gather gene expression profiles and information from BC patients. Next, using m7G‐regulated differentially expressed genes (DEGs), we created a predictive multigene signature that was confirmed by qRT‐PCR. Moreover, we identified potential key regulators and experimentally validated the underlying mechanism. Finally, we used functional enrichment analysis to study the main processes. The overview of the design and main findings of the current study is displayed in Data S1.

## 2 METHOD

### 2.1 Data collection

The TCGA database was used to retrieve the RNA sequence data (RNA‐seq) as well as patient medical records of BC. The ‘limma’ R package's scaling method was used to standardize gene expression profiles.^11^ The read count numbers were normalized. The TCGA data are accessible to the public. Consequently, clearance from regional ethics committees was not required. In this study, we adhere to the TCGA's criteria for data access and dissemination. Then, 29 m7G‐related genes were found in prior publications^12^ and the gene list of m7G modification in GSEA public datasets (http://www.gsea‐msigdb.org/gsea/login.jsp), which are included in Data S2.

### 2.2 Establishing and confirming a prognosis m7G‐regulated gene signature

DEGs from tumour organs and nearby healthy tissue (FDR <0.05) were identified using the ‘limma’ R program. The univariate Cox regression analysis of overall survival (OS) was performed to determine whether the genes or the model are significantly predictive in m7G. *p*‐value was adjusted using the Benjamini and Hochberg method (BH). Networks of interacting DEGs (version 11.0) were constructed in the STRING database. LASSO method was used to build a prognosis model and reduce the possibility of overfitting via the processes of factor selection and shrink with the function of the ‘glmnet’ R package.^13^ Dependent variables included OS and clinical status, and the standardized expression matrix of predictive DEGs was the independent variables in the regression. Using minimum criterion, tenfold cross‐validations were used to determine the model's penalty parameter. Patient's risk scores were calculated using the normalized amounts of mRNA, as well as the related regression coefficients. The following formula was devised:

$$\text{Risk score}\;\left(\text{patients}\right)=\sum_{\begin{array}{c} i=1 \end{array}}^{n}\text{coefficient}\;\left(\text{gene}i\right)\times \text{expression value}\;\left(\text{gene}i\right).$$

Based on the median level of the risk score, the samples were classified into low‐ and high‐risk groups. Based on the expression matrix of genes in the signature, PCA was done utilizing the ‘prcomp’ technique of the ‘stats’ package. The finest expression cut‐off value for each gene's survival analysis was established using the ‘survival’ R program. To verify the gene signature's predictive value, time‐dependent ROC curve analysis was performed using the ‘timeROC’ R package.

### 2.3 Functional enrichment analysis

DEGs were conducted with functional enrichment analysis of GO and KEGG analysis (|log2FC| > 1, FDR < 0.05) using ‘clusterProfiler’ package in R.^14^ The *p*‐values were modified using the BH method. The activity of 13 immune‐related pathways and the grade of invasion of 16 immune cells were evaluated using ssGSEA via the ‘gsva’ package. The file for the annotation gene set can be found in Data S3. To identify the biological functional differences between the two risk groups, gene set variation analysis (GSVA) was conducted.

### 2.4 Tumour mutation burden analysis

The total number of somatic mutations (excluding silent mutations) for each tumour sample was referred to as the tumour mutation burden (TMB). The ‘tmb’ function of the maftools R package calculated the TMB value for each sample.

### 2.5 Correlation of NCBP1 with immune features

The prognostic values of the four genes utilized to build the risk model were evaluated using Kaplan–Meier and ROC analyses. NCBP1 was found to have excellent predictive efficiency. GSEA was performed to explore the potential mechanism underlying the function of NCBP1 in BC. For GSEA analysis, the R packages ‘enrichplot’, ‘clusterProfiler’, ‘limma’ and ‘org.Hs.eg.db’ were utilized. To investigate the correlation between the level of NCBP1 and the enrichment scores of several types of immunity cells, we used the CIBERSORT algorithm. We further analysed the relationships between the expression levels of NCBP1 and immune checkpoint genes and visualized the results in a heatmap.

### 2.6 Statistical analysis

To assess gene expression between cancer samples versus neighbouring normal tissues, a Student's *t*‐test was conducted. To compare proportional differences, the chi‐squared test was performed. The Mann–Whitney test with *p* values adjusted by the BH approach was performed to evaluate the ssGSEA scores of immune cells or pathways between the different levels of risk groups. Using Kaplan–Meier analysis, the log‐rank test was utilized to compare the OS of different groups. Both uni‐ and multivariate Cox regression analyses were used to identify independent factors of OS. All statistical calculations were performed using R software (Version 3.5.3). Unless otherwise noted, a *p*‐value of 0.05 or less was considered statistically significant.

### 2.7 Cell lines and cell culture

Hunan Fenghui Biotechnology Co., Ltd. provided the normal mammary epithelial cell line MCF10A and the BC cell lines MDA‐MB‐231. Beijing Biosynthesis Biotechnology Co., Ltd. provided the BC cell lines MCF‐7. MDA‐MB‐231 and MCF‐7 cells were grown in Dulbecco's modified Eagle's medium with 10% (v/v) fetal bovine serum (Gibco) and 1% penicillin–streptomycin. MCF10A cells were cultured in DMEM/F12 media with 5% (v/v) FBS, 20 ng/mL EGF, 10 g/mL insulin and 500 ng/mL hydrocortisone. These cell lines were maintained at 37°C in a 5% CO_2_ atmosphere.

### 2.8 Analyses using the quantitative real‐time polymerase chain reaction

Total RNA was isolated from BC cells using the TRIzol reagent. A NanoDrop 2000 spectrophotometer was used to determine the concentration and purity of total RNA (Thermo Fisher Scientific, Wilmington, DE, USA). The MonScriptTM RTllll Super Mix with dsDNase kit created complementary DNA (Monad). The following conditions were used to run quantitative real‐time PCR using MonAmpTM ChemoHS qPCR Master Mix: 95°C for 10 min, followed by 40 cycles at 95°C for 10 s, 55°C for 30 s. Table 1 contains the primer sequences for detection. GAPDH expression was employed as an internal standard control. The 2^−△△Ct^ technique was used to gather and quantify all gene expression levels. Three separate experiments yielded the findings.

**TABLE 1:** Primers for qRT‐PCR.

Gene name	Sequence (5′–3′)
GAPDH	F: 5′‐TGACTTCAACAGCGACACCCA‐3′ R: 5′‐CACCCTGTTGCTGTAGCCAAA‐3′
EIF4E	F: 5′‐CTGCGGCTGATCTCCAAG‐3′ R: 5′‐TTCCCACATAGGCTCAATACC‐3′
EIF4E3	F: 5′‐AAGGGTGGCGTATGGAAGATGAAAG‐3′ R: 5′‐CGGTCCCGAACACTGACACTAAC‐3′
NCBP1	F: 5′‐GCTAAAGAGAAACTTGCTAGGC‐3′ R: 5′‐GGTCAAGATCATGATAAACCGC‐3′
WDR4	F: 5′‐AGAGTTTGTGAGCCGTATCTC‐3′ R: 5′‐GAAGATGTAGACCACAGGAGTG‐3′

### 2.9 Western blotting

The protein was extracted with western blot (WB) lysis buffer (BMP2020, Abbkine) containing PMSF and quantified with a BCA kit (SW101‐02, Severn Biotech). An equal amount (20 μg) of protein extracts was separated on 10% SDS‐PAGE gels then transferred to PVDF membranes (Immobilon‐P, Millipore). The cell membrane was treated with the appropriate initial antibodies at 4°C overnight after being blocked in 5% non‐fat milk for 1.5 h. After washing three times with TBST for 10 min, the membranes were incubated with the secondary antibody (1: 5000 dilution; Biosharp, China) for 2 h at room temperature. After washing with TBST for half an hour, the signals were visualized using ECL reagent (BMU102‐CN, Abbkine, China) and quantified using Image Lab Software (Bio‐Rad). Primary antibodies were listed as follows: EIF4E (AB33766, Abcam), NCBP1 (10349‐1‐AP, Proteintech), WDR4 (PAB40170, Bioswamp), GAPDH (BM1623, Boster Biological Technology Co. ltd).

### 2.10 siRNA interference assay

NCBP1 siRNA and scrambled siRNA were purchased from IBSBIO (Shanghai, China). A scrambled siRNA served as a contrast. The siRNA sequences were as follows (Table 2): siNCBP1‐1: GGUAUGGACUGCUGAUAAACC, siNCBP1‐2: GGAUGAUGACGACGAUGAAGG, siNCBP1‐3: GGAAGAAGCUAAAGAGAAACU and siRNA‐NC: UUCUCCGAACGUGUCACGUTT. According to the manufacturer's instructions, the siRNAs were transfected into BC cells using Lipofectamine 2000 (Invitrogen). Total RNA was extracted 48 h after transfection, and protein was extracted 72 h after transfection. RNA and protein were estimated, respectively by quantitative real‐time polymerase chain reaction (RT‐PCR) and WB.

**TABLE 2:** siRNA sequences for knockdown NCBP1.

siRNA	siRNA sequences
NCBP1‐643	Sense: 5′‐GGUAUGGACUGCUGAUAAACC‐3′
	Anti‐sense: 5′‐UUUAUCAGCAGUCCAUACCUG‐3′
NCBP1‐1585	Sense: 5′‐GGAUGAUGACGACGAUGAAGG‐3′
	Anti‐sense:5′‐UUCAUCGUCGUCAUCAUCCUG‐3′
NCBP1‐1975	Sense: 5′‐GGAAGAAGCUAAAGAGAAACU‐3′
	Anti‐sense: 5′‐UUUCUCUUUAGCUUCUUCCAG‐3′
siRNA‐NC	Sense: 5′‐UUCUCCGAACGUGUCACGUTT‐3′
	Anti‐sense: 5′‐UUUCUCUUUAGCUUCUUCCAG‐3′

### 2.11 CCK‐8 assay

After MDA‐MB‐231 cells were transfected with NC and siNCBP1 for 24 h, they were respectively seeded into 96‐well culture plates (5 × 10^3^/well). Each will need to add DMEM medium (100 μL). The cell proliferation was measured using a Cell Counting Kit‐8 at 0, 24, 48 and 72 h. 10 μL of CCK8 solution was added to each well, and cells were incubated for 2 h at 37°C at these points. Finally, a microplate reader was used to measure the absorbance of each well.

### 2.12 Cell migration and invasion assays

FBS‐free cell suspensions were seeded in a transwell chamber (Corning, Kennebunk, ME, USA) (1–2 × 10^4^/well) coated with Matrigel (invasion) or without Matrigel (migration). The lower well was supplemented with a medium containing 20% FBS. After incubation for 48 h, cells across the membrane were fixed with 4% paraformaldehyde for 20 min and then treated with crystal violet dye for 10 min. The cells in the upper chamber were gently removed with a cotton swab. Cells were photographed and counted under a microscope.

## 3 RESULTS

### 3.1 Identify prognostic m7G‐related DEGs

**FIGURE 1:**
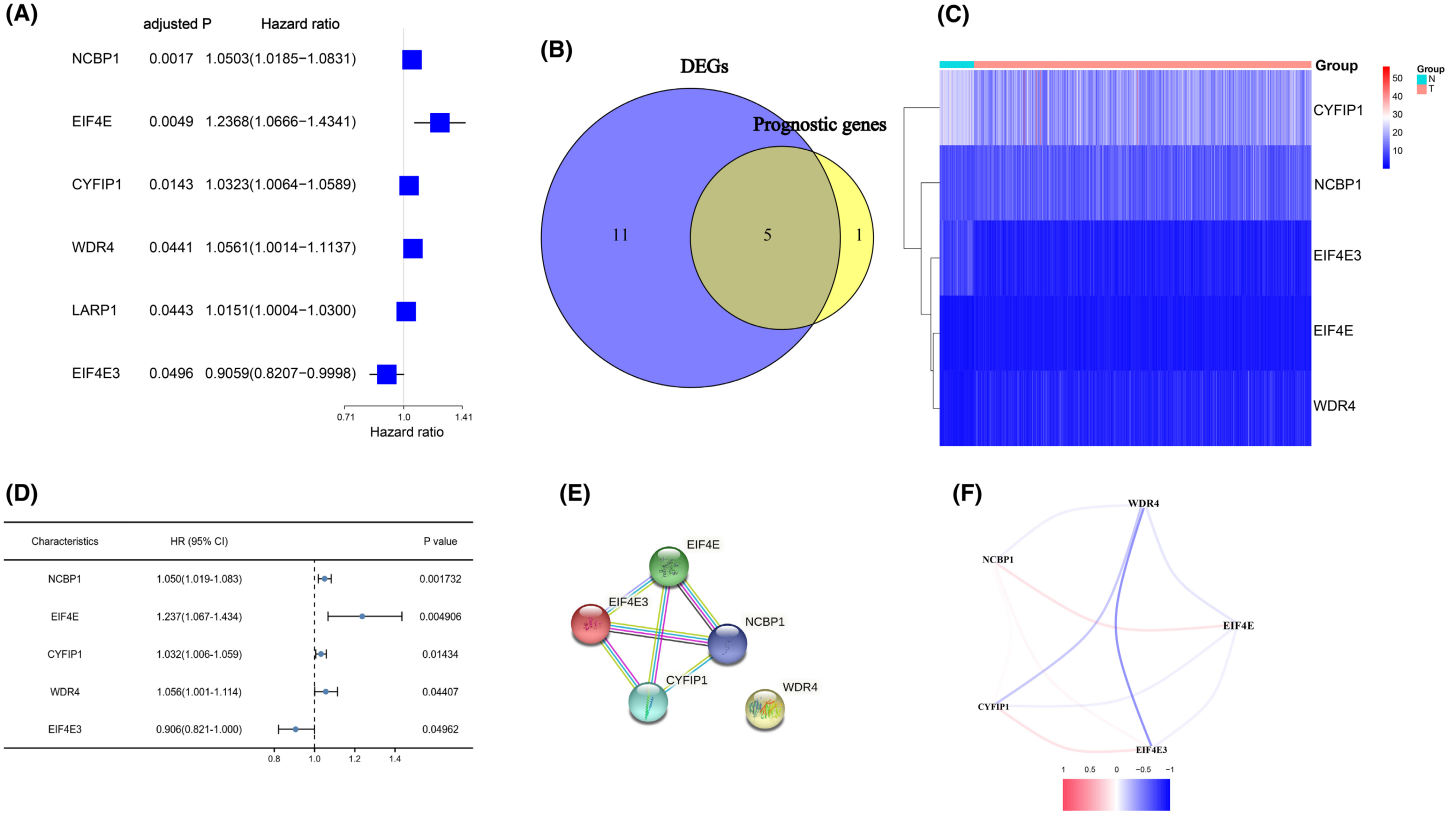
The candidate m7G‐related genes have been identified. (A) Forest plots displaying the findings of the univariate cox regression analysis of 6 genes with *p* < 0.05. (B) A Venn diagram was used to identify genes that were not only differently expressed between the tumour and neighbouring normal tissue but also were associated with overall survival (OS). (C) In tumour tissue, all five overlapping genes were elevated. (D) Forest plots displaying the findings of the univariate Cox regression analysis of gene expression versus OS. (E) The interactions between the candidate genes were highlighted by the PPI network obtained from the STRING database. (F) The candidate gene correlation network. Different hues show the correlation coefficients.

The majority of m7G‐related genes (16/29, 55.2%) were found to vary between tumour and nontumorous tissues (Table 3). In the univariate cox regression analysis, six genes have been shown to be linked with OS among 29 m7G‐related genes (Figure 1A). Finally, five m7G‐related genes in total are not only expressed differently but they were also linked to OS (FDR < 0.05, Figure 1B–D). The interaction network among these genes revealed that EIF4E, EI4EF3, NCBP1 and CYFIP1 are all strongly related (Figure 1E). Figure 1F depicts the relationship between these genes.

**TABLE 3:** The significant results of differently expressed analysis among m7G‐related genes.

Number	Gene	logFC	FDR
1	CYFIP1	−0.43883829	9.37E‐33
2	DCPS	0.563991753	7.42E‐15
3	EIF4E	0.345914628	7.31E‐13
4	EIF4E1B	2.740602283	1.85E‐06
5	EIF4E2	0.192549034	1.23E‐06
6	EIF4E3	−1.115893709	4.45E‐43
7	EIF4G3	−0.177080175	1.02E‐07
8	GEMIN5	−0.211412734	2.37E‐09
9	LSM1	0.604553678	2.84E‐09
10	METTL1	0.722795603	1.96E‐26
11	NCBP1	0.24329601	2.91E‐08
12	NCBP2	0.168526178	3.27E‐05
13	NCBP3	−0.269352253	2.43E‐17
14	NUDT16	−0.657372405	4.58E‐40
15	NUDT3	0.335225767	4.22E‐12
16	WDR4	0.792631846	9.58E‐26

### 3.2 Establishing and validating a prognostic model

**FIGURE 2:**
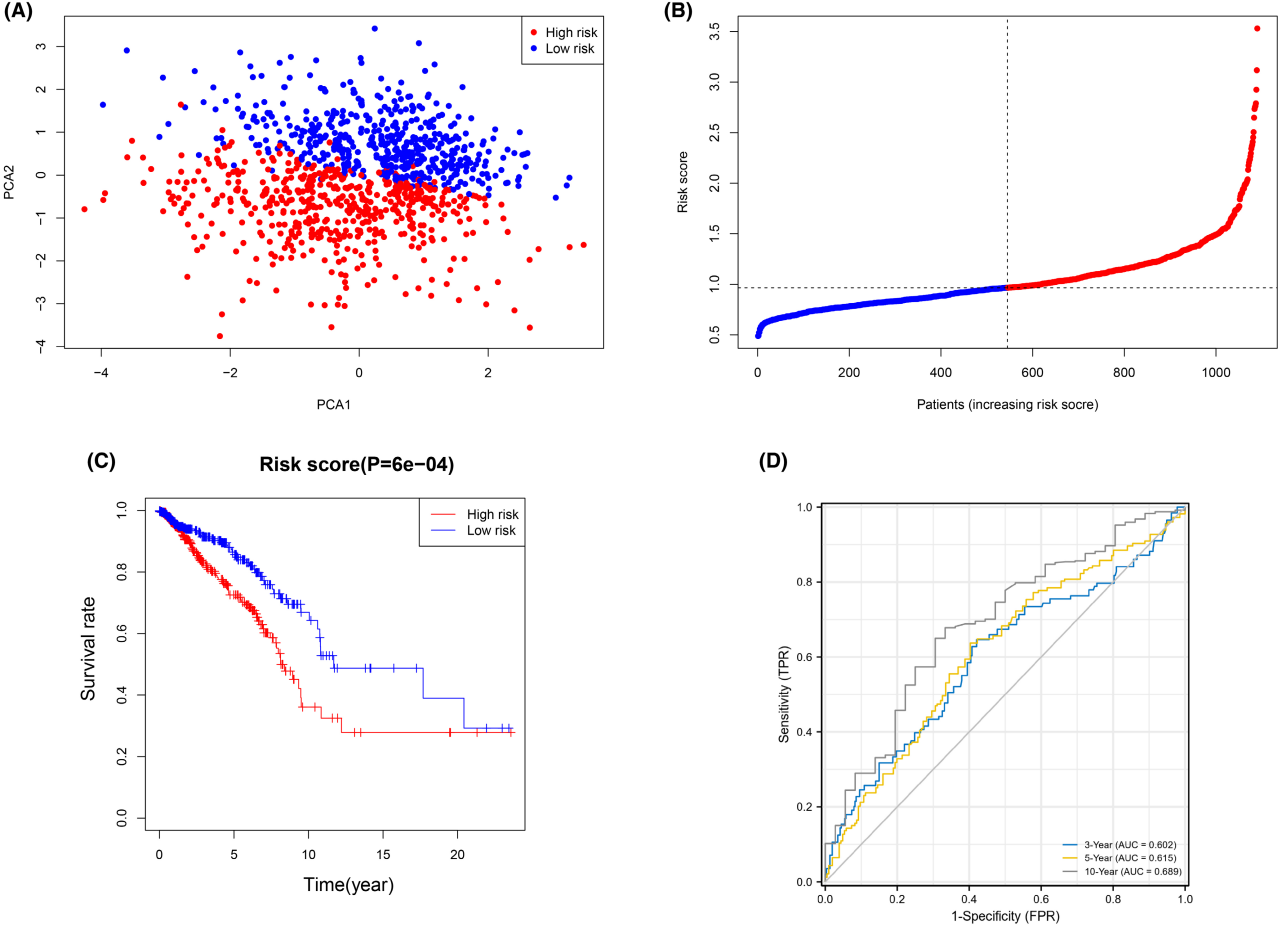
Prognostic analysis of the four‐gene signature model. (A) The distribution and median value of the risk scores. (B) PCA plot of the breast cancer expression matrix. (C) The distributions of overall survival status, and risk score. (D) The risk score's prognostic ability was confirmed using the AUC of time‐dependent ROC curves.

The expression patterns of the five genes mentioned above were used in LASSO analysis to create a prognostic model. Based on the optimum value, a four‐gene signature was discovered (Data S4). The risk score was calculated using the formula shown below: score = 1.2215 × EIF4E expression level + 0.9397 × EIF4E3 expression level + 1.0390 × NCBP1 expression level + 1.0333 × WDR4 expression level. The patients were split into two groups based on their median threshold values: high‐ and low‐risk groups (each group number = 545) (Figure 2A). According to PCA, patients in distinct risk categories were dispersed in two directions (Figure 2B). Individuals in the high‐risk group had a significantly lower life expectancy than their low‐risk counterparts, according to the Kaplan–Meier curve (Figure 2C, *p* < 0.001). The AUC for the risk score for OS was 0.602 after 3 years, 0.615 after 5 years, and 0.689 after 10 years, according to time‐dependent ROC curves (Figure 2D).

### 3.3 The four‐gene signature's important predictive value

To confirm if the risk score was an unbiased predictive factor of OS, researchers ran cox analysis (univariate and multivariate) on the relevant factors. The risk score was strongly linked with OS in univariate Cox regression models in the TCGA cohorts (HR = 1.9472, 95% CI = 1.3408–2.8279, *p* = 0.0005) (Data S5). When additional confounding factors were excluded from the Cox regression (multivariate) analyses, the risk score maintained an independent prognostic factor of OS (HR =2.1272, 95% CI = 1.4529–3.1143, *p* < 0.001).

### 3.4 Functional analysis

DEGs of low and high groups were applied in GO and KEGG pathway analysis to discover associated mechanisms and pathways associated with risk scores. Surprisingly, several biological processes and KEGG pathways associated with neuroactive ligand–receptor interaction were clearly enhanced in the DEGs (*p* < 0.001, Data S6).

**FIGURE 3:**
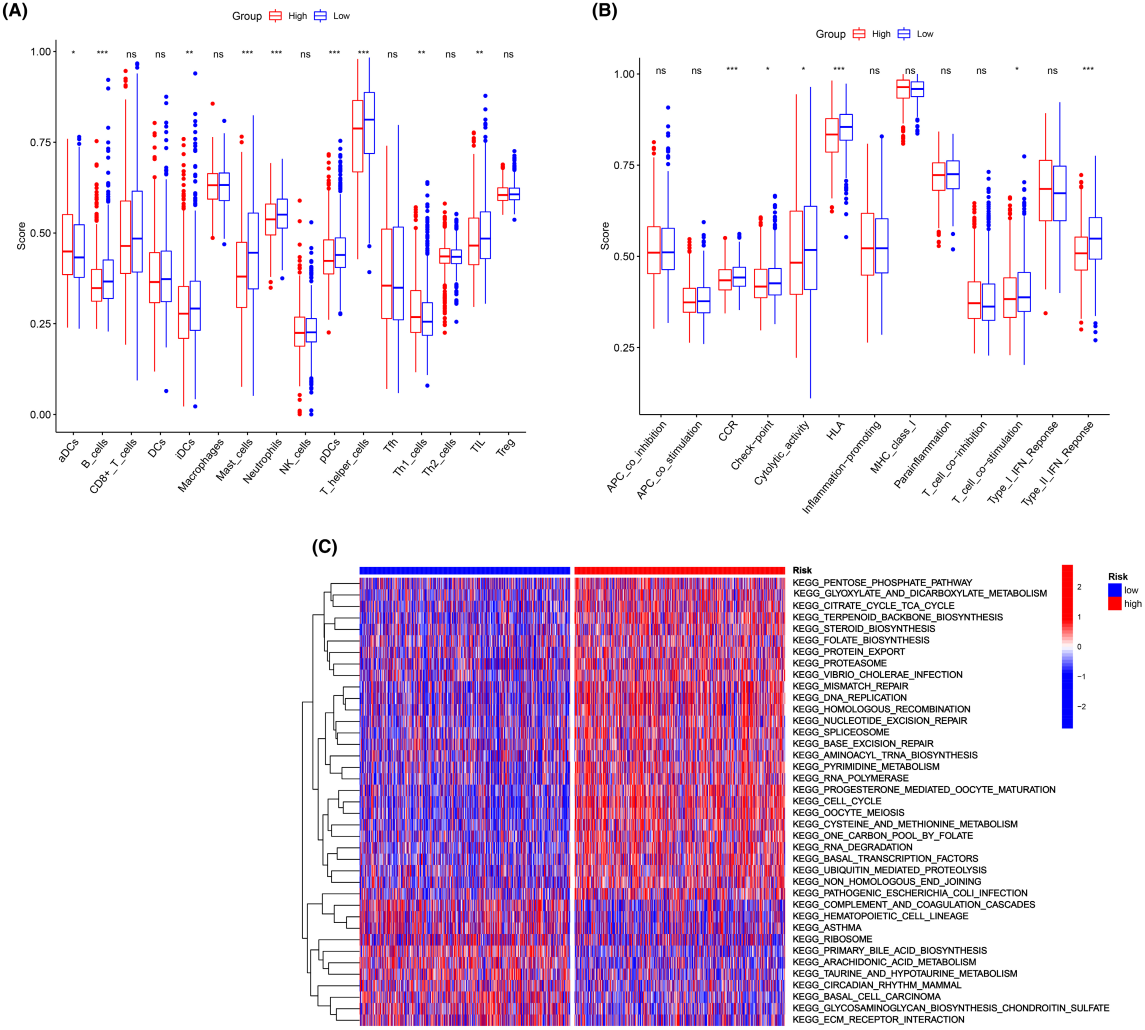
Analysis of ssGSEA scores over risk groups. Boxplots depict the scores for 16 immune cells (A) and 13 immunological‐related activities (B). (C) GSVA of KEGG pathways between high‐ and low‐risk groups. ns, not significant; *p < 0.05; **p < 0.01; ***p < 0.001.

We employed ssGSEA to calculate the enrichment scores of various immune cell subpopulations, associated functions, and pathways in order to examine the correlation between the risk score and the immunological state. Significant differences were observed in the contents of the antigen presentation process, including neutrophil, B cells, DCs, mast cells, Th1 cells and T helper cell scores, among the groups (*p* < 0.001, Figure 3A). Within the low‐risk individuals, higher scores were observed for the Type II IFN response and HLA (*p* < 0.001, Figure 3B). We then utilized the GSVA package to investigate the KEGG pathways associated with the risk score (Figure 3C). These pathways, which are involved in nucleic acid metabolism and protein metabolism, include the pentose phosphate pathway, citrate cycle, terpenoid backbone biosynthesis, and steroid biosynthesis.

### 3.5 Examining the frequency of gene mutations and the chromosomal copy number variation in BC patients

**FIGURE 4:**
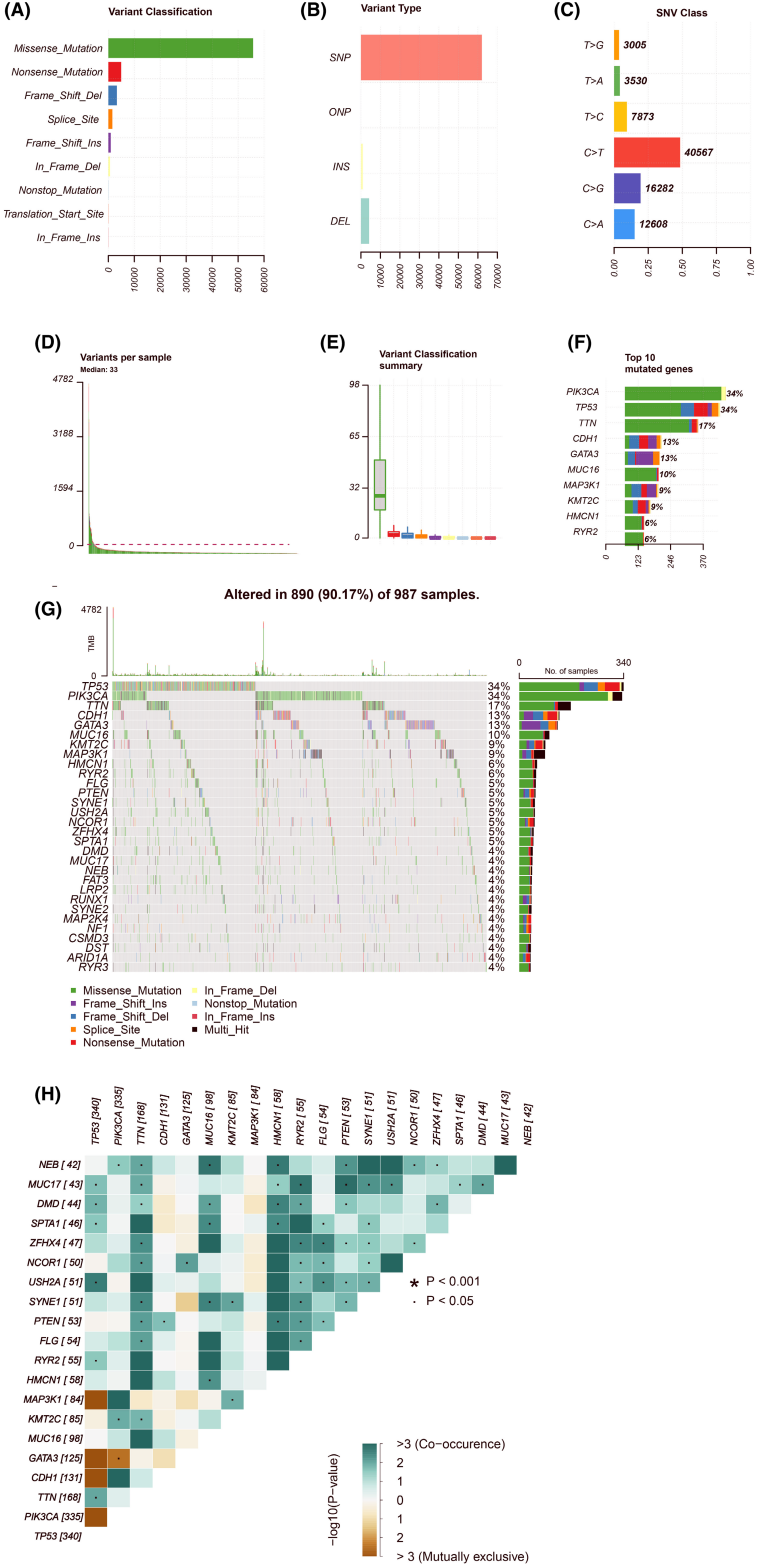
Panoramic view of breast cancer mutations in TCGA dataset. (A–F) Mutation panorama analysis; (G) top 30 gene mutation waterfall map; (H) The coincident and exclusive relationship among mutated genes.

Upon completing the process of filtering away samples with missing information taken from the TCGA dataset, we obtained mutation information of BC patients, which we used to investigate the effects of mutations for these patients. First, we tried to analyse the mutation profile of BC patients, finding that missense mutations accounted for the vast majority, that SNPs were the most common type of variant, and that C>T was the most common type of SNP in patients with BC (Figure 4A–C). We obtained the number of base changes for each patient and distinguished the mutation types with different colours in the box plot (Figure 4D,E). PIK3CA (34%), TP53 (34%), TTN (17%), CDH1 (13%), GATA3 (13%), MUC16 (10%), MAP3K1 (9%), KMT2C (9%), HMCN1 (6%) and RYR2 (6%) were the top five genes with the highest mutation frequencies in BC (Figure 4F). The top 30 mutant genes for each patient are specifically listed in Figure 4G. Figure 4H reveals the relationship between these genes.

### 3.6 Relationship between four‐gene signature and drug sensitivity

We investigated the IC_50_ values of 20 chemotherapeutics between the groups at high and low risk. The result showed that IC_50_ values of imatinib, lenalidomide, masitinib, mitomycin C, navitoclax, pyrimethamine, thapsigargin, tivozanib, veliparib, AICAR, epothilone B and gefitinib of the high‐risk group were less than those of low‐risk group, indicating that patients in the high‐risk group were more likely to benefit from these 20 drugs (Data S7).

### 3.7 Survival analysis of the four genes in breast cancer

**FIGURE 5:**
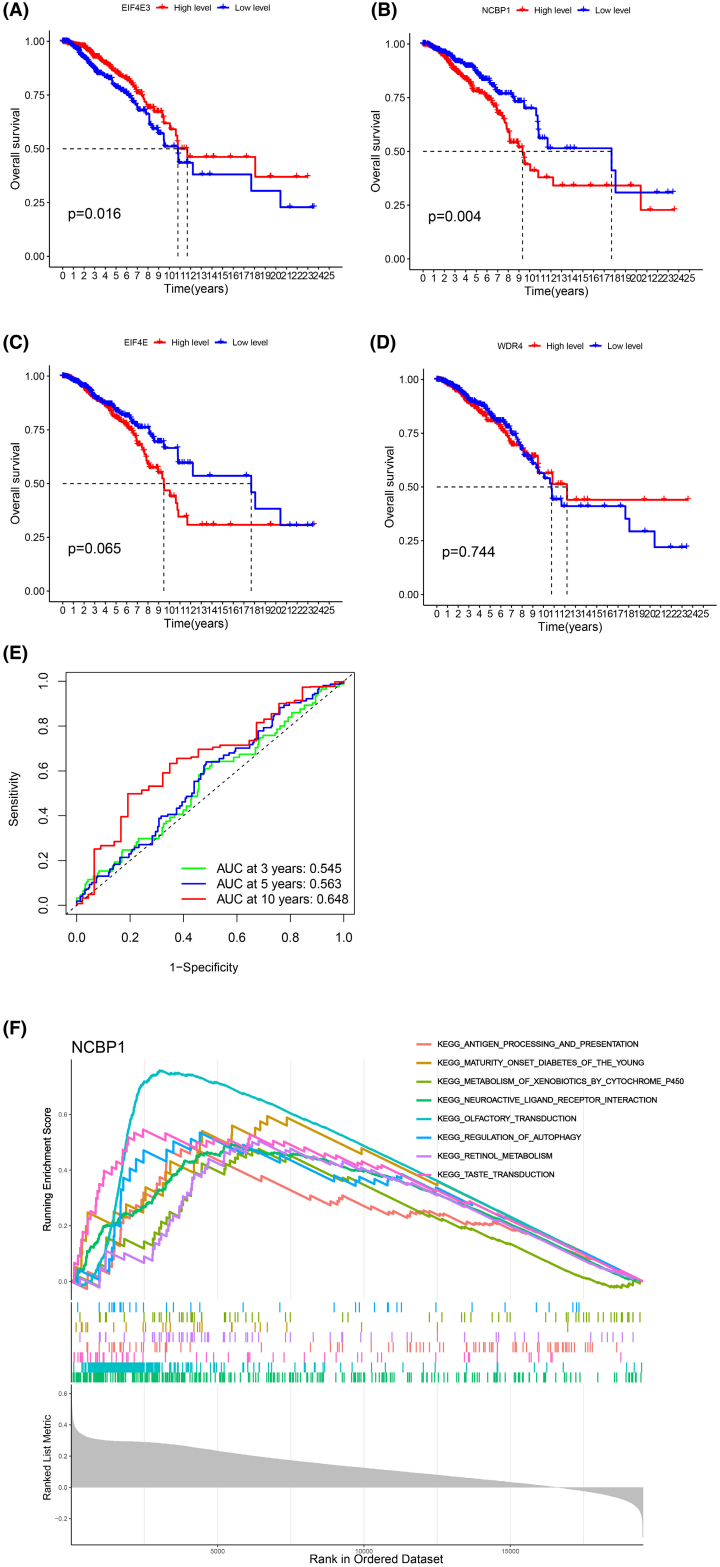
(A–D) Overall survival (OS) of the four genes in patients with breast cancer was analysed by Kaplan–Meier plotter. EIF4E3, *p* = 0.016; NCBP1, *p* = 0.004; EIF4E, *p* = 0.065; WDR4, *p* = 0.744. (E) ROC plots of NCBP1. (F) GSEA analysis of NCBP1.

OS analyses of the four genes (EIF4E, EIF4E3, NCBP1, WDR4) used to construct the model were further conducted by the Kaplan–Meier plotter. As demonstrated in Figure 5A–D, the high expression level of NCBP1 in BC patients was associated with poor OS (*p* = 0.004), while the high expression level of EIF4E3 in BC patients was associated with better OS (*p* = 0.016). Moreover, according to the ROC result of other genes, NCBP1 had better 3‐, 5‐ and 10‐year prognosis in BC patients (Figure 5E, Data S8). To investigate the potential function of NCBP1, we performed a GSEA analysis (Figure 5F). The result shows that the function of NCPB1 in BC is mainly enriched in the neuroactive interaction between ligand and receptor and transduction.

### 3.8 Relationship between NCBP1 and tumour immune microenvironment

**FIGURE 6:**
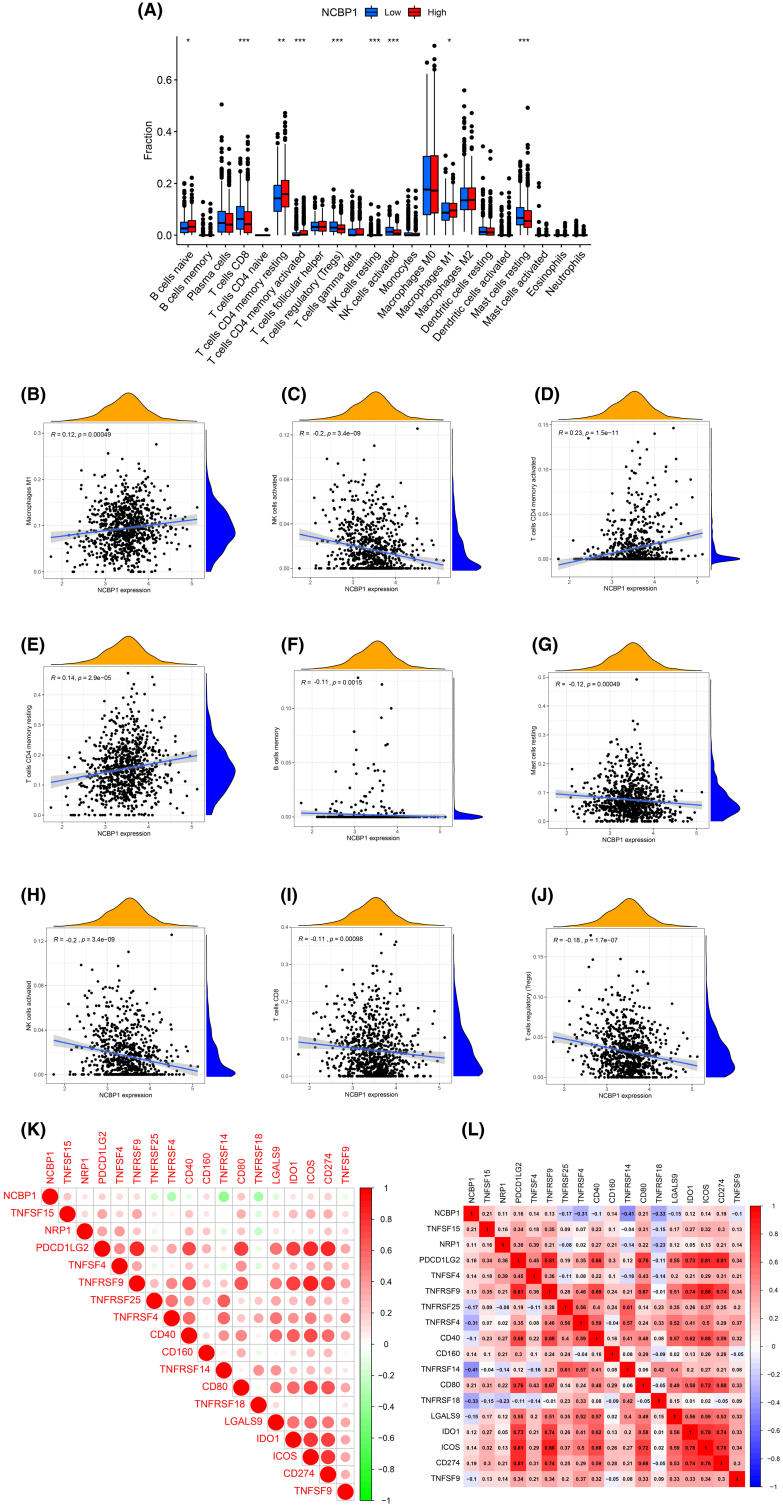
(A) Changes of 22 immune cell subtypes between high and low NCBP1 expression groups in breast cancer (BC) samples. (B–J) Correlation between the expression of NCBP1 and immune infiltrating cells in BC. (K, L) Correlations between NCBP1 and immune checkpoints.

To investigate the relationship between NCBP1 expression and cells infiltrating the immune system in BC, we employed the ssGSEA method and the ‘limma’ R package. The findings demonstrated a correlation between the expression of NCBP1 and the infiltration of immune cells in the tumour (Figure 6A), including naive B cells, CD8 T cells, CD4 memory resting T cells, CD4 memory activated T cells, regulatory T cells, NK cells resting and activated NK cells, macrophages M1 and resting mast cells. NCBP1 has a positive correlation with the expression levels of macrophages M1 (*r* = 0.12, *p* = 0.00049), NK cells resting (*r* = 0.11, *p* = 0.0022), T cells CD4 memory activated (*r* = 0.23, *p* = 1.5e‐11) and T cells CD4 resting (*r* = 0.14, *p* = 2.9e‐05), while negatively correlated with the expression levels of B cell memory (*r* = −0.11, *p* = 0.0015), mast cells resting (*r* = −0.12, *p* = 0.00049), NK cells activated (*r* = −0.2, *p* = 3.4e‐09), T cells CD8 (*r* = −0.11, *p* = 0.00098) and T cells regulatory (*r* = −0.18, *p* = 1.7e‐07) (Figure 6B–J). Figure 6K,L shows the relationship between NCBP1 and immunity checkpoints. We found that the expression levels of TNFSF15, NRP1, PDCD1LG2, TNFSF4, TNFRSF9, CD160, CD80, IDO1, ICOS and CD274 correlated strongly to NCBP1, while TNFRSF25, TNFRSF4, CD40, TNFRSF14, TNFRSF18, LGALS9 and TNFSF9 were negatively associated with the expression level of NCBP1. In conclusion, NCBP1 expression might be closely associated with the reprogramming of the immunosuppressive microenvironment in BC.

### 3.9 Identification of NCBP1 critical role in BC

**FIGURE 7:**
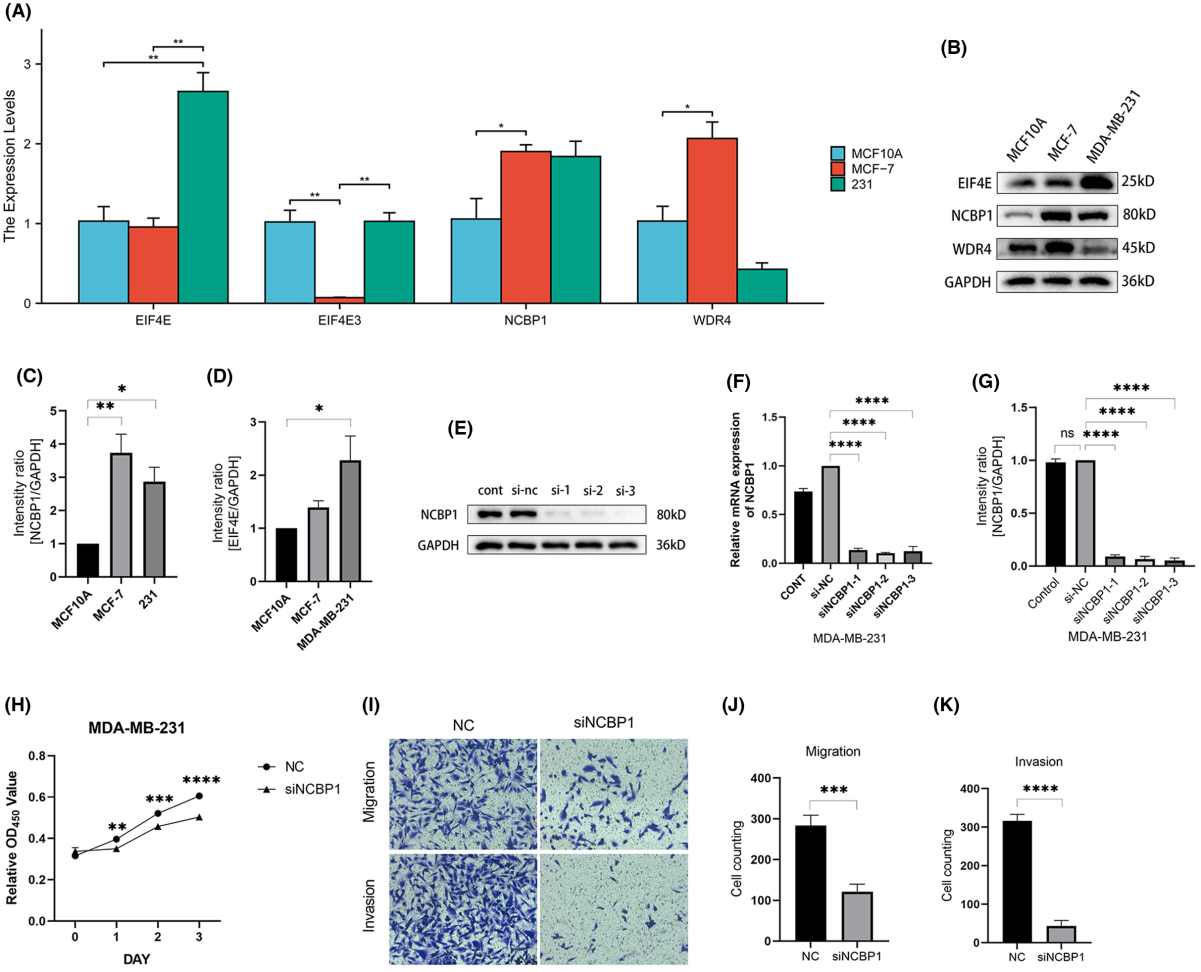
Hub genes expression level in breast cancer (BC) cell lines and the epithelial cell line. (A) Expression level of EIF4E3, EIF4E, NCBP1 and WDR4 in tumour and normal cells. Green represents 231 BC cells. Red presents MCF‐7 BC cells. Blue represents MCF10A normal endothelial cells. (B–D) Western blot analysis for the hub genes in 231 BC cells, MCF‐7 BC cells and MCF10A normal endothelial cells. (E–G) NCBP1 expression was verified after transfection in NCBP1. (H) Proliferation assays revealed attenuated capabilities of 231 after knocking down NNCBP1. (I–K) Migration and invasion abilities of 231 were weakened after knocking down NCBP1. *p < 0.05; **p < 0.001.

To better confirm the importance of NCBP1 in BC, researchers also confirmed the expression of four hub genes in normal mammary epithelial cell line MCF10A, the BC cell line MDA‐MB‐231, and the BC cell line MCF‐7. The findings revealed that the expression of NCBP1 was increased in MDA‐MB‐231 and MCF‐7, with MCF‐7 showing a statistically significant difference (*p* < 0.05) (Figure 7A). The findings of WB also demonstrated that MB‐231 and MCF‐7 expressed themselves greatly more than did typical endothelial cells (Figure 7B,C). EIF4E is also highly expressed significantly in the MDA‐MB‐231 cell line, yet not in MCF‐7 (Figure 7D).

### 3.10 NCBP1 promotes proliferation, migration and invasion of BC

NCBP1 dysregulation in BC suggested that NCBP1 might influence the progression of BC. To test this hypothesis, we successfully constructed 231 cell lines with knocked‐down NCBP1 by transfecting siRNA (Figure 7E–G). CCK‐8 assays were used to assess the proliferation abilities of BC cells. As shown in Figure 7H, the proliferation rate of 231 cells was significantly repressed when knocking down NCBP1 levels. Transwell assays were performed to assess cell migration and invasion abilities. It was obvious that migration and invasion capabilities were suppressed in NCBP1 knocked‐down cells (Figure 7I–K).

## 4 DISCUSSION

Although some researchers^15^, ^16^, ^17^ have suggested that various genes may influence m7G in BC, their relationship with BC patients' OS remained uncertain. The current work explored the expression level of 29 m7G‐regulated genes in BC tissue and their relationships with OS. Surprisingly, the majority (55.2%) of m7G‐related genes were differentially expressed between tumour and normal samples, and five genes were associated with OS in Cox regression analysis. These findings strongly suggested that m7G may have a role in BC, as well as the ability to develop a prognostic model using these m7G‐regulated genes. Then one new predictive model including four m7G‐regulated genes was developed and identified in a separate population. Analysis of enrichment function revealed that the mechanism of hub genes is associated with immune pathways. Moreover, we discovered a strong correlation between nuclear cap binding protein 1 (NCBP1) and the prognosis of BC patients. Further explorations on the relationship among NCBP1 and the tumour's microenvironment revealed that NCBP1 was significantly associated with several kinds of specific immunity cells like T cells or non‐specific immunity like macrophages, and many immune checkpoints, providing a potential direction for the treatment of BC.

Cell viability and poly (A) RNA output require NCBP1 and nuclear cap binding protein 2 to form a nuclear cap binding complex (CBC), which opens the flow of genetic information from DNA to protein.^18^, ^19^ In higher eukaryotes, NCBP1 and another nuclear cap binding protein NCBP3 together form another CBC, which binds to the components of the m RNA processing mechanism and promotes the output of poly (A) RNA.^20^ It binds to the m7G′‐cap structure of freshly transcribed mRNA and coordinates downstream RNA biosynthesis processes such as nucleoplasm transport and recruitment of translation factors in the cytoplasm.^21^, ^22^ The abnormal expression of NCBP1 was observed in lung adenocarcinoma and diffuse large B‐cell lymphoma.^23^, ^24^

Meanwhile, EIF4E3 and EIF4E also exert an important effect on the development of BC based on our survival analysis. EIF4E3 (The translation initiation factor 4E), a member of the EIF4E family of translation initiation factors, interacts with the 5′‐cap structures of mRNA.^25^ In the eukaryotic protein translation initiation machinery, EIF4E (eukaryotic translation initiation factor 4) is a critical part of the protein known as the translation initiation factor complex. EIF4E recognizes 7‐methylguanosine‐containing caps on mRNA and binds to them, releasing the mRNA's secondary structure and so facilitating ribosome interaction at the initiation of protein synthesis. eIF4E expression and activation are linked to cell transformation and the tumour starts as a proto‐oncogene. Specific RNA binding proteins and microRNAs, as well as influencing the 5‐cap binding activity of EIF4E, may selectively modulate mRNA transcript translation.^26^, ^27^ Previous research has revealed that individuals who had increased EIF4E expression are more likely to recur and die compared to those who had reduced eIF4E expression in triple‐negative BC patients.^28^ EIF4E3 is a member of the EIF4E family of proteins that bind to the 5′‐cap structure of messenger RNA.^27^, ^29^ According to one research, EIF4E3 depends on cap‐binding activity to operate as a tumour suppressor and compete with EIF4E's growth‐promoting capabilities. In fact, tumours that have increased EIF4E and low EIF4E3 expression demonstrate that EIF4E3 is of essential inhibitory mechanism that is lost in certain cancers.^30^ Other investigations have shown that EIF4E3 may prevent oncogenic transformation.^31^, ^32^, ^33^ WDR4, a member of the WD repeat protein family, plays a crucial role in various aspects of cell development including cell cycle progression, signal transduction, gene regulation, apoptosis and more.^8^, ^34^, ^35^ Studies have shown that WDR4 plays a crucial role in the development of malignant tumours, the aberrant expression of WDR4 is closely associated with tumour immunity and tumour mutation load.^36^

While the mechanisms behind tumour sensitivity to m7G have been extensively studied, the potential impact of m7G on tumour immunity remains elusive. We used the DEGs to do a GO enrichment analysis, which showed significant enrichment for a wide variety of immune‐related biological functions and pathways across all of the different risk categories. Especially, antigen presentation pathway components varied significantly from low‐risk groups to high‐risk groups in this study. Previous research has shown that Ribavirin would compete with EIF4E to join with m7G, hence limiting mRNA transcription and having a function in tumour therapy.^37^ It can directly improve tumour immunotherapy by targeting these methylation‐regulating molecules. Using a bioinformatics tool to evaluate the database, it was shown that several modulators had a substantial link with tumour immune responses and immunotherapy in nasopharyngeal cancer, BC, and pancreatic cancer. Furthermore, the greater risk score was related to poorer antitumor immunity, especially type II IFN response activity. As a result, reduced immunity against tumours in high‐risk individuals may explain their poor prognosis.

Through analysing the co‐expression association between immune checkpoint genes and NCBP1 in TCGA datasets, our work elucidated that NCBP1 and various immune checkpoint genes have a positive correlation in BC. Furthermore, there exists a correlation between the expression of NCBP1 and the infiltration of immune cells in adrenal cancer and osteosarcoma. Hence, despite NCBP1's function as a cap‐binding complex facilitating molecular into the cell matrix, our hypothesis assumes that NCBP1 might potentially have a significant influence on immunological dysfunction in BC. In this work, an investigation was conducted to analyse the biological function of NCBP1 using GO analysis. The results revealed that NCBP1 plays a crucial role in inflammatory activities and immunological responses in BC. Furthermore, the utilisation of both the CIBERSORT algorithm and ssGSEA algorithm revealed that the overexpression of NCBP1 was associated with increased infiltration of naive B cells, NK cells, Macrophages M1, mast cells, regulatory T cells, and various subtypes of activated memory CD4^+^ and CD8 T cells. The increased infiltration level of immune cells in BC induces tumour cells to upregulate many immunological checkpoints as a defence mechanism against the immune system. Hence, a strong upregulation of NCBP1 was shown to have a significant association with the infiltration of immune cells and was indicative of a reduced OS in patients diagnosed with BC.

This study possesses several limitations. First, the established prognostic model was developed and validated using historical datasets, necessitating stronger evidence to ascertain its clinical value. Additionally, the inherent limitation of relying on a single predictor marker to construct the prognostic model was unavoidable, potentially resulting in the exclusion of crucial prognostic genes in BC. Furthermore, it is important to highlight that the relationship between the risk score and immunological activity remains unexplored experimentally. In conclusion, the research identified a new predictive model based on four m7G‐related genes. This model was shown to be independently linked with OS, offering insight into BC prognosis prediction. The fundamental processes underlying the relationship between m7G‐related genes and tumour immunity in BC are yet unknown and deserve additional exploration.

## AUTHOR CONTRIBUTIONS

**Wenqi Bai:** Conceptualization (equal). **Jianrong Li:** Methodology (equal). **Lin Zheng:** Data curation (equal). **Liying Song:** Formal analysis (equal). **Zhuanxia Dong:** Project administration (equal). **Liqiang Qi:** Funding acquisition (equal).

## CONFLICT OF INTEREST STATEMENT

The authors confirm that there are no conflicts of interest.

## Supporting information


Data S1.

Data S2.

Data S3.

Data S4.

Data S5.

Data S6.

Data S7.

Data S8.

## Data Availability

Data available in article supplementary material.
