# Supplementary figures and images for: Identification and validation of N 7‐methylguanosine‐associated gene NCBP1 as prognostic and immune‐associated biomarkers in breast cancer patients
Source: J Cell Mol Med. 2023 Dec 10;28(2):e18067. doi: 10.1111/jcmm.18067 (PMC10826432; doi:10.1111/jcmm.18067)

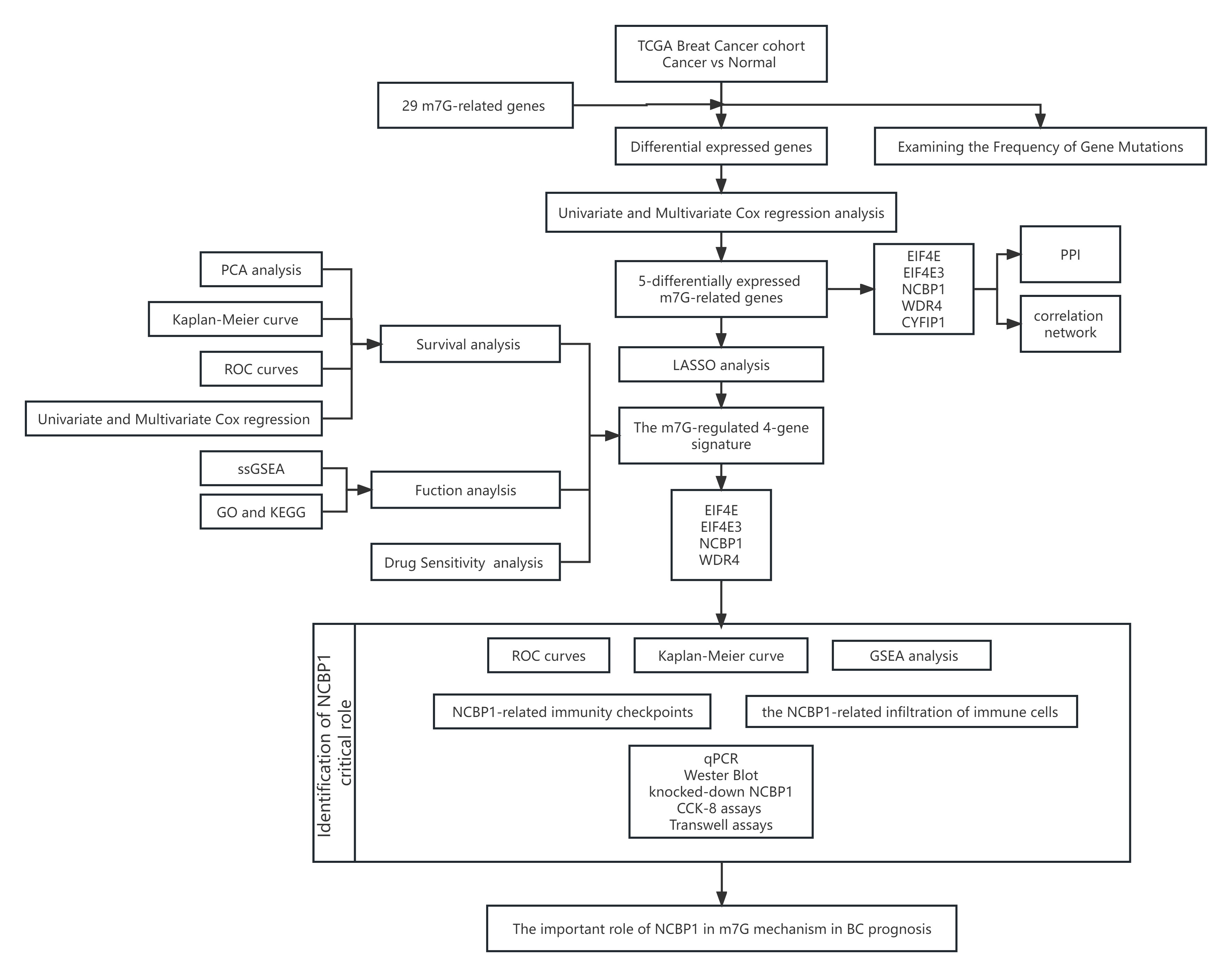

Supplement: Supplementary file 1 — Data S1. [file JCMM-28-e18067-s005.jpg]

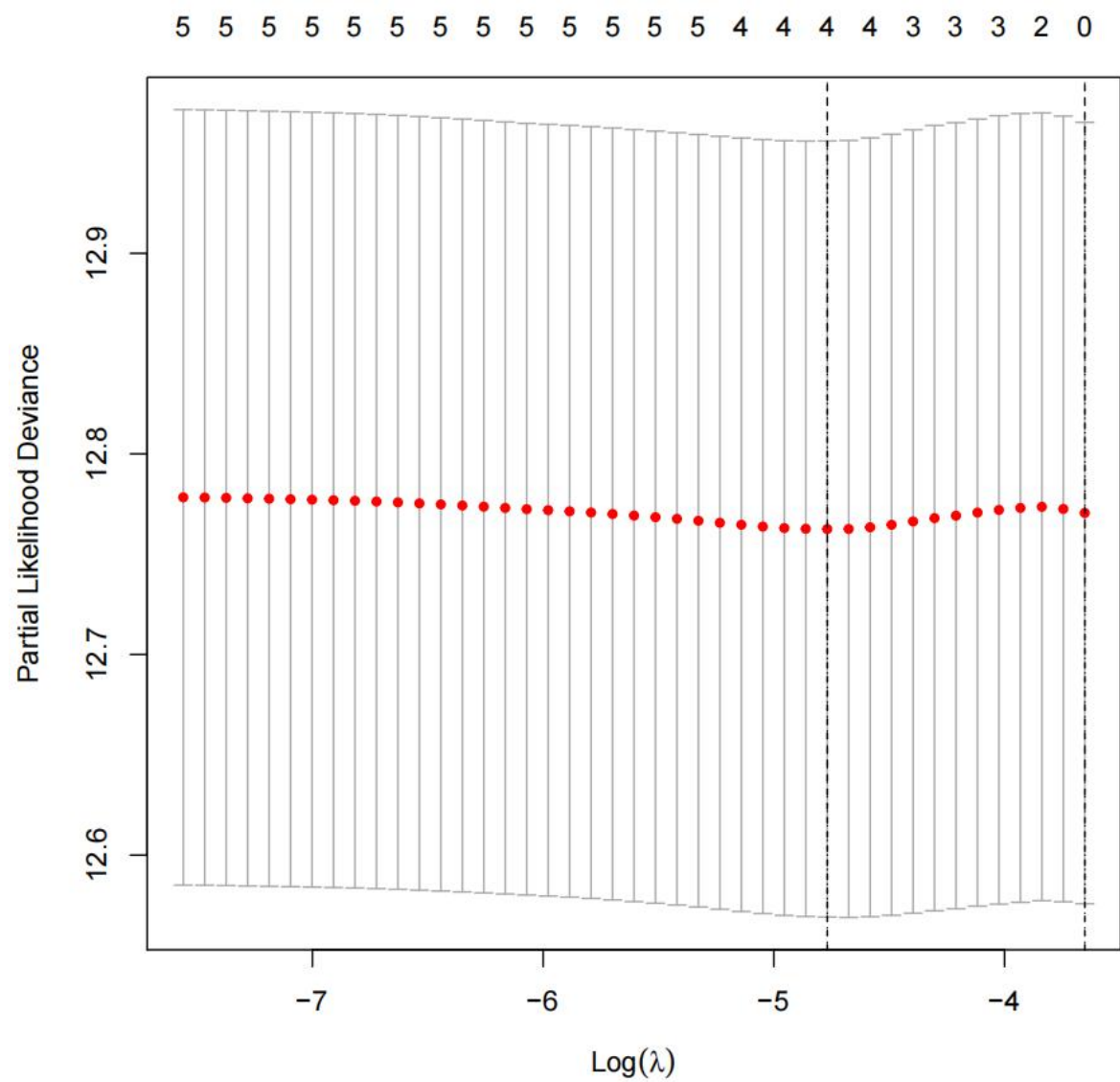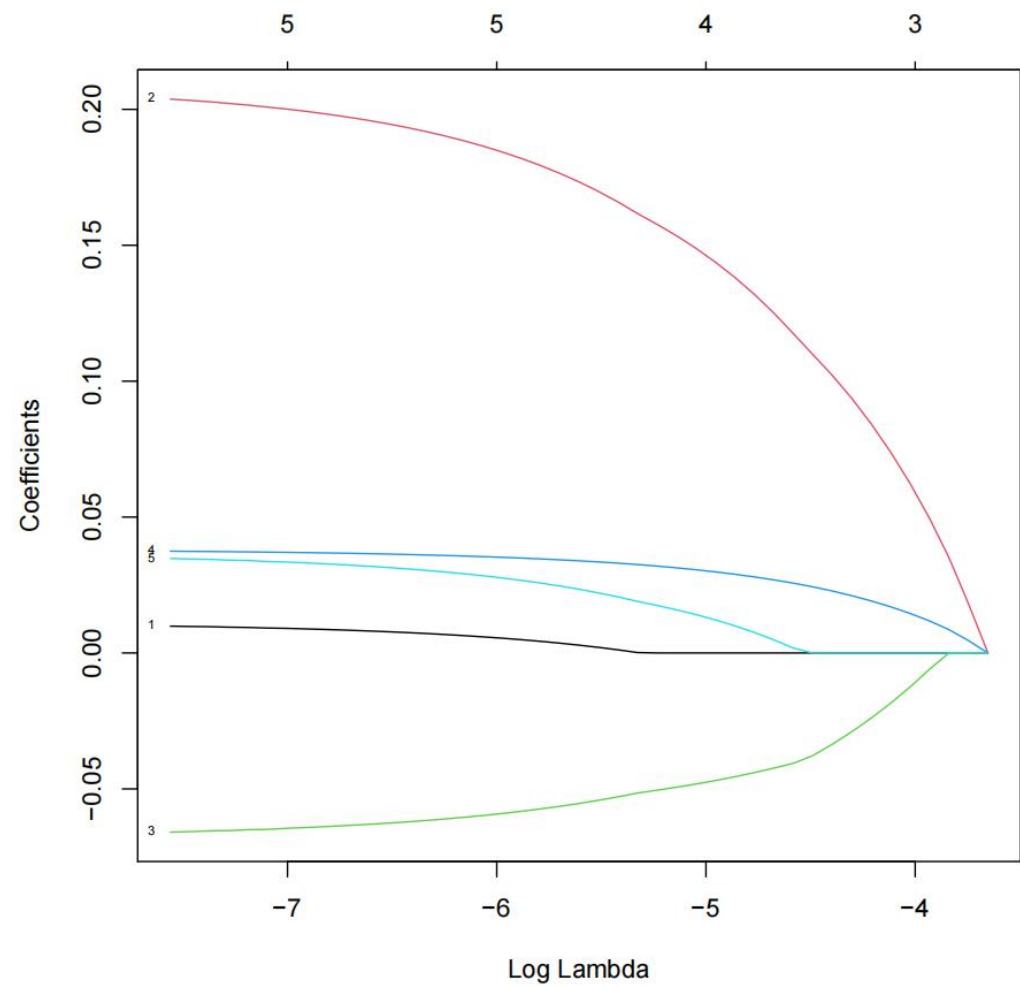

Supplement: Supplementary file 4 — Data S4. [file JCMM-28-e18067-s008.pdf]

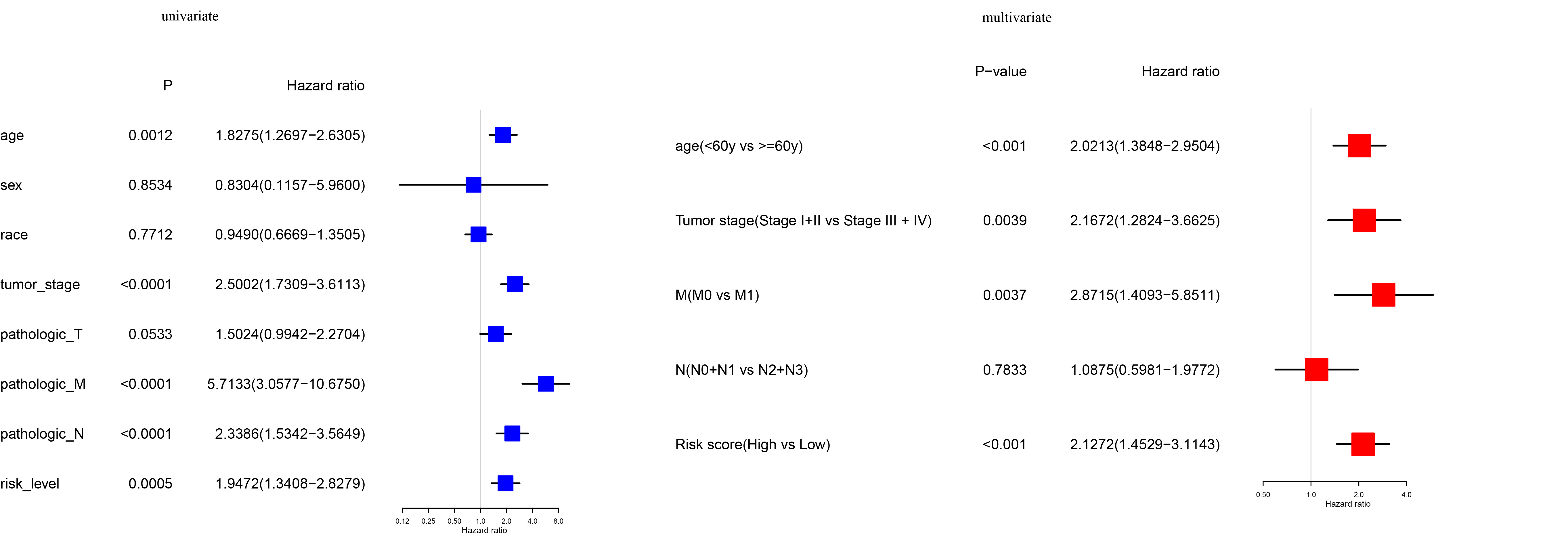

Supplement: Supplementary file 5 — Data S5. [file JCMM-28-e18067-s001.jpg]

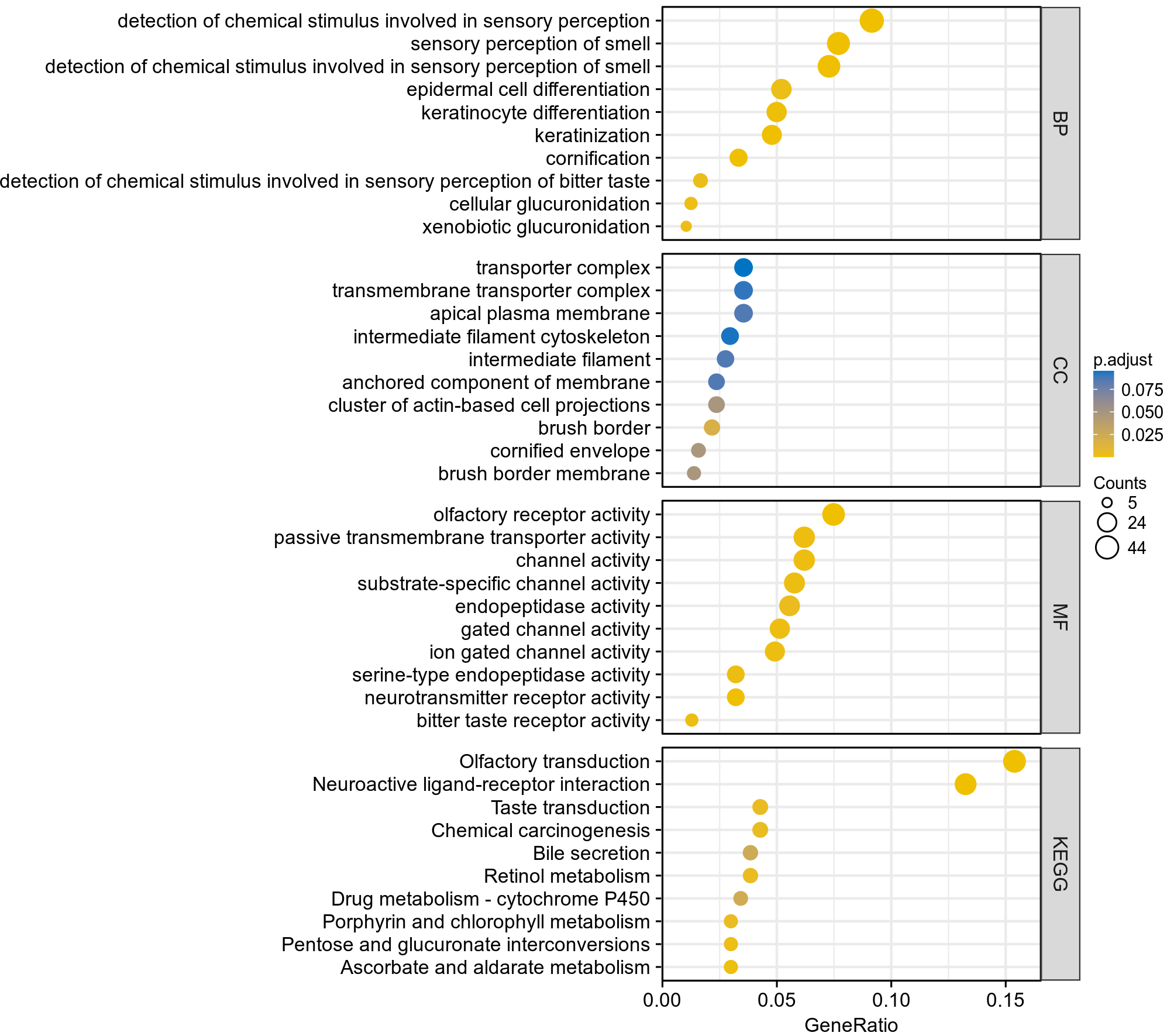

Supplement: Supplementary file 6 — Data S6. [file JCMM-28-e18067-s007.tiff]

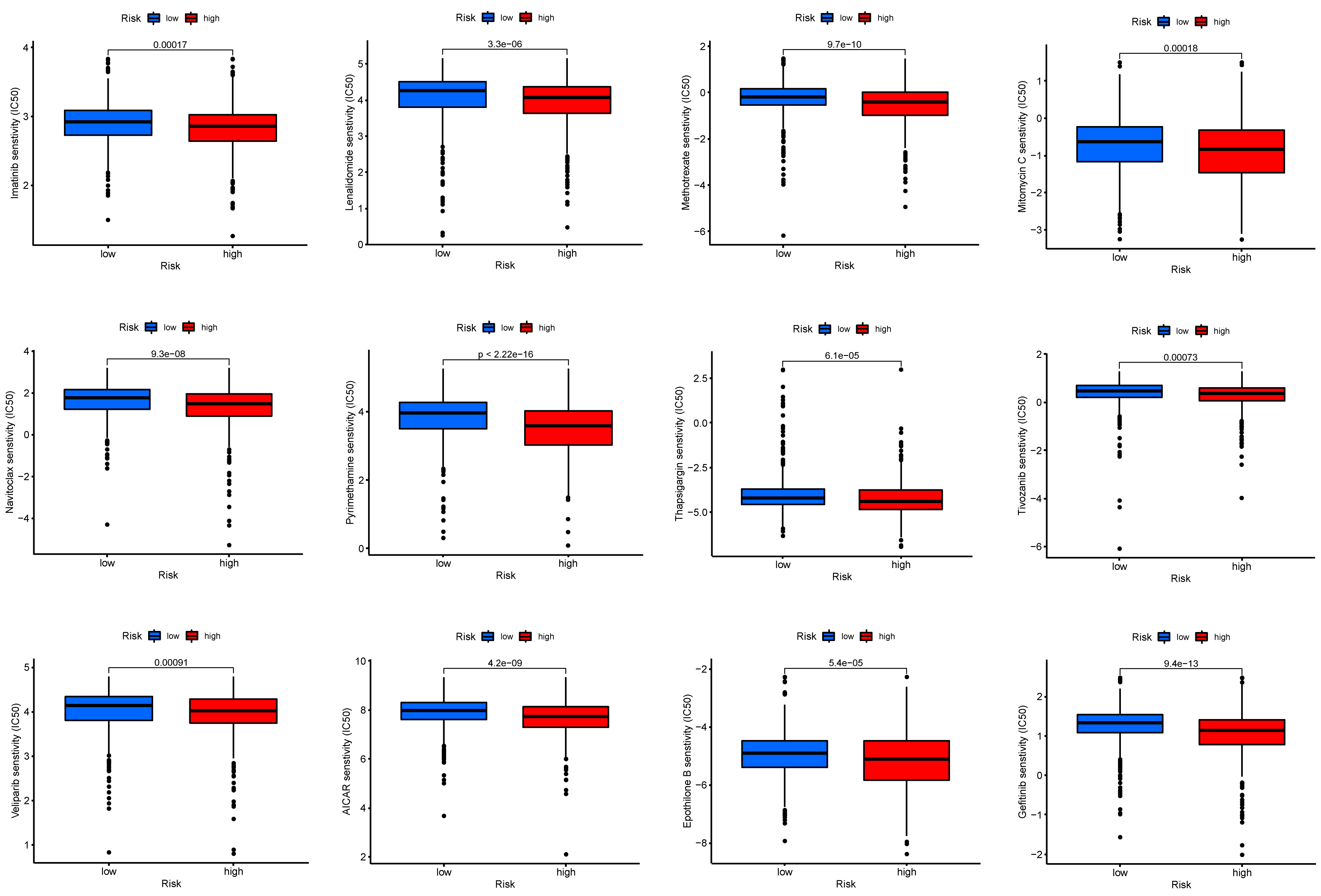

Supplement: Supplementary file 7 — Data S7. [file JCMM-28-e18067-s003.tif]

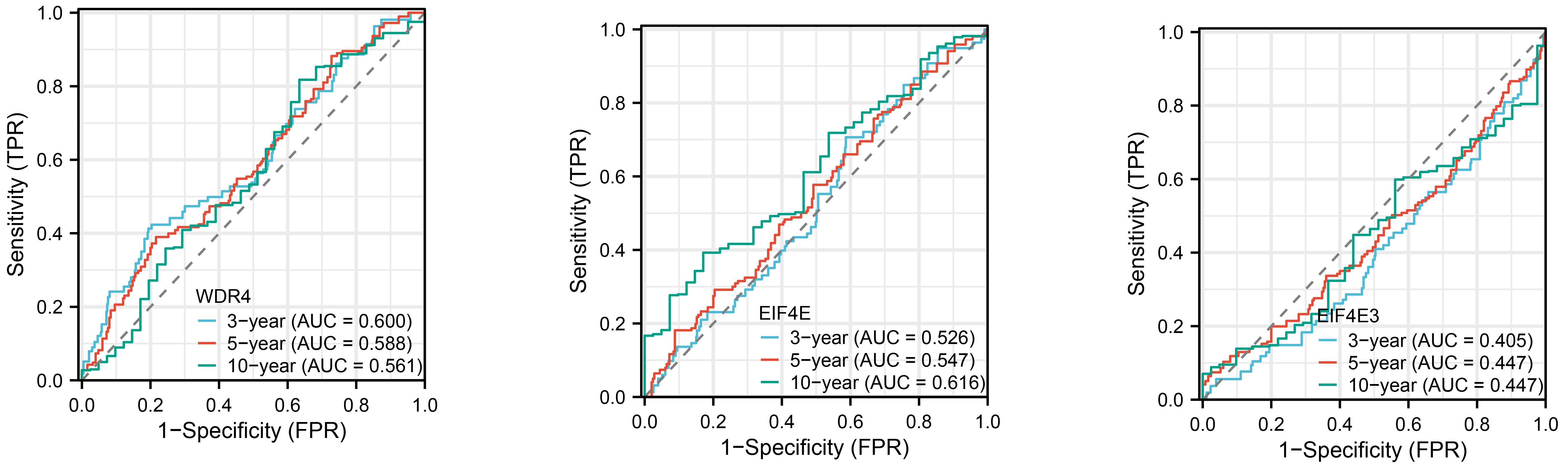

Supplement: Supplementary file 8 — Data S8. [file JCMM-28-e18067-s002.jpg]
